# Study on the prevalence of diabetic retinopathy in Pudong New Area and the predictive value of estimated glucose disposal rate: a cross-sectional study

**DOI:** 10.3389/fendo.2025.1571488

**Published:** 2025-06-25

**Authors:** Jiaojiao Gao, Kang Wu, Xiaonan Wang, Zhitao Li, Xiaolin Liu, Jiahui Song, Yang Liu, Hua Qiu, Feng Wei, Qian Xu, Shaotan Xiao, Yi Zhou, Xiaonan Ruan

**Affiliations:** Shanghai Pudong New Area Center for Disease Control and Prevention (Shanghai Pudong New Area Health Supervision Institute), Pudong Institute of Preventive Medicine of Fudan University, Shanghai, China

**Keywords:** diabetic retinopathy, prevalence, insulin resistance, estimated glucose disposal rate, predictive value

## Abstract

**Objective:**

To describe the prevalence of diabetic retinopathy (DR) in patients with type 2 diabetes mellitus (T2DM) in Pudong New Area, Shanghai, and to explore the association between estimated glucose disposal rate (eGDR) and DR, as well as the possibility of eGDR as a predictive marker.

**Methods:**

Field investigation was conducted among sampled patients with T2DM from May to September 2023. Fundus examination was performed by automatic mydriatless fundus camera to evaluate the prevalence of DR. Restricted cubic spline (RCS) function was employed to evaluate the dose-response relationship between eGDR and DR. Logistic regression analysis was performed to examine their association.

**Results:**

A total of 2,327 patients with T2DM completed the survey, with a median age of 69 years. The prevalence of DR in Pudong New Area was 7.39%, and RCS curve demonstrated a significant negative linear relationship between eGDR and DR. After controlling for confounding factors, the results of the logistics regression showed that the risk of DR decreased by 40.4% for every one standard deviation increase in eGDR. The area under the ROC curve for eGDR predicting DR was 0.777 and 0.781, indicating a high predictive value of eGDR for DR.

**Conclusion:**

There is a significant negative linear association between eGDR and the risk of DR, and eGDR is expected to serve as a target for DR prediction and intervention.

## 1 Introduction

Diabetic retinopathy (DR) is the most common form of diabetic microangiopathy, with a global prevalence of approximately 22.27% (1). It has become the leading cause of blindness among the working-age population due to the visual impairment caused by diabetic macular edema (2). The analysis of the fundus disease spectrum revealed a significant increase in the prevalence of DR among individuals under 35 years of age between 2019 and 2021 (3). Previous research indicated patients with type 2 diabetes mellitus (T2DM) exhibited suboptimal adherence to recommended eye examinations, and a study conducted in Shanghai found that the average awareness level of DR in T2DM was only 12.37%, and the rate of fundus examination was only 12.34% (4, 5). The early symptoms of DR are insidious, with gradual pathological changes, making early detection and intervention particularly challenging. Thus, early fundus examination and research into the potential influencing factors of DR hold significant clinical value for enhancing the management quality of T2DM patients and preventing vision loss due to retinopathy.

Insulin resistance (IR) is a typical pathological change in T2DM, and studies have shown that IR significantly increases the risk of cardiovascular disease (6). Insulin measurement is invasive and costly, and recently some IR surrogate markers based on non-insulin detection have been developed. The estimated glucose disposal rate (eGDR), as an emerging indicator, is considered a reliable substitute for IR. Studies suggest that eGDR can serve as an intervention target to mitigate the risk of macrovascular disease, including cardiovascular events (7, 8). However, the relationship between eGDR and microvascular diseases, such as DR, is limited, and the association may be modified by gender and other characteristics (9, 10). Therefore, this study aims to analyze the current epidemiological status of DR among patients with T2DM in Pudong New Area, and to investigate the association between eGDR and DR, as well as the predictive value of eGDR for DR.

## 2 Materials and methods

### 2.1 Participants

A multi-stage stratified random cluster sampling method was employed to conduct a sampling survey on T2DM from May to September 2023. The inclusion criteria were: T2DM and living in Pudong New Area for more than 6 months. The exclusion criteria were: (1) Type 1 diabetes, gestational diabetes mellitus, and special types of diabetes; (2) Complications of acute diabetes, severe cardiovascular and cerebrovascular diseases, malignant tumors, and stress states; (3) Patients who have undergone vitrectomy or retinal laser photocoagulation; (4) Patients with unclear fundus images (9, 10). The sample size calculation formula for the current study is: $n=\frac{pq}{{\left(d/z_{a}\right)}^{2}}$ , where p is the expected prevalence rate. Referring to previous studies, p was taken as 0.22 (1), α was set to 0.05, at which point z_α_ was 1.96, and the common allowable error was 0.15p. Considering non-response bias, an additional 20% sample size was added, thus, the calculated sample size was approximately 736. The sample size of this survey meets the requirements.

### 2.2 Methods

#### 2.2.1 Questionnaire

The self-designed questionnaire was determined by literature review and expert review, including (1) basic information: gender, age, marital status, education, family history, tobacco and alcohol use, living situation, sleep situation, physical activity; (2) blood sugar management and control: time of initial diabetes diagnosis, history of hypoglycemia in the past year, complications of diabetes.

#### 2.2.2 Anthropometric measurements and assessments of glycosylated hemoglobin

(HbA1c) **levels** Height, weight, waist circumference, hip circumference, and blood pressure were measured on site and accurately recorded. HbA1c levels were measured using boronate affinity chromatography (brand: Sinocare, model: PCH-50) based on solid-phase immunoassay methodology, which determined the percentage of HbA1c relative to total hemoglobin.

#### 2.2.3 Fundus examination

A fully automatic non-mydriatic fundus camera (brand: Airdoc, model: AI-FD16aF) was used for fundus examination of T2DM. DR was staged into non-proliferative DR and proliferative DR according to *Evidence-based guidelines for diagnosis and treatment of diabetic retinopathy in China* (2022) (11). Each eye was photographed separately, and if the severity of the lesions in both eyes was different, the more severe side was used for staging.

#### 2.2.4 Sleep status survey

The Pittsburgh Sleep Quality Index (PSQI) was utilized to assess the sleep quality of participants over the past month. The total score ranges from 0 to 21, where a higher score signifies poorer sleep quality, and a score exceeding 7 indicates clinically significant sleep disturbances (12).

#### 2.2.5 Assessment of cognitive function

The Montreal Cognitive Assessment Basic Scale (MoCA-B) (13) was utilized, with a maximum score of 30. For participants with different levels of education, the cutoff scores for Mild Cognitive Impairment (MCI) were set as follows: ≤ 19 for those with ≤ 6 years of education, ≤ 22 for those with 7–12 years of education, and ≤ 24 for those with more than 12 years of education.

#### 2.2.6 Physical activity survey

The World Health Organization’s Global Physical Activity Questionnaire (GPAQ) was utilized to evaluate participants’ physical activity levels across work, transportation, and recreational domains, as well as their daily sedentary time (14). A metabolic equivalent of task score of 600 or higher per week indicated that the physical activity level met the recommended standard. According to self-reports, sitting or reclining for a total of more than 480 minutes per day was considered severe sedentary behavior (15).

#### 2.2.7 Calculation of eGDR

The calculation formula for eGDR (mg/(kg·min)] is as follows: eGDR = 21.158 - [0.09 × waist circumference (cm)] - [3.407 × hypertension (yes/no)] - [0.551 × HbA1c (%)] (9). A lower eGDR indicates a diminished capacity of the body to process glucose and a higher degree of insulin resistance.

#### 2.2.8 Ethical approval

This study was approved by the Medical Ethics Committee of Pudong New Area Center for Disease Control and Prevention, Shanghai (Approval No. PDCDCLL-20220718-001), and written informed consent was obtained from all participants.

### 2.3 Quality control

All investigators involved in the field investigation are certified practicing physicians from primary-level medical institutions. Prior to the investigation, all investigators undergo standardized training. Additionally, at least two personnel from the Centers for Disease Control and Prevention concurrently conduct quality control during each investigation.

### 2.4 Statistical methods

Data cleaning and statistical analysis were performed using SPSS 23.0 and SAS 9.1.3 software. Normality of continuous variables was assessed using the Kolmogorov-Smirnov test. Normally distributed measurement data were described using mean ± standard deviation (SD), while skewed distribution data were characterized by median (interquartile range). For normally distributed data with homogeneous variance, one-way ANOVA or independent two-sample t-tests were employed for comparisons. Chi-square tests were used for categorical data comparisons. Restricted cubic splines (RCS) were employed to evaluate the dose-response relationship between eGDR and DR. Logistic regression analysis was performed to examine their association, with Model 1 remaining unadjusted and Model 2 incorporating adjustments for confounding factors including family history, history of hypoglycemia, sleep disorders, mild cognitive impairment, and disease duration. The predictive performance of eGDR for DR was subsequently assessed through receiver operating characteristic (ROC) curve analysis. The eGDR values were categorized into three groups based on percentile distribution. Subgroup analyses were performed to confirm the robustness of the results (8, 9). All statistical tests were conducted at a significance level of α=0.05.

## 3 Results

### 3.1 Epidemiological status of DR in Pudong New Area

**Figure 1:**
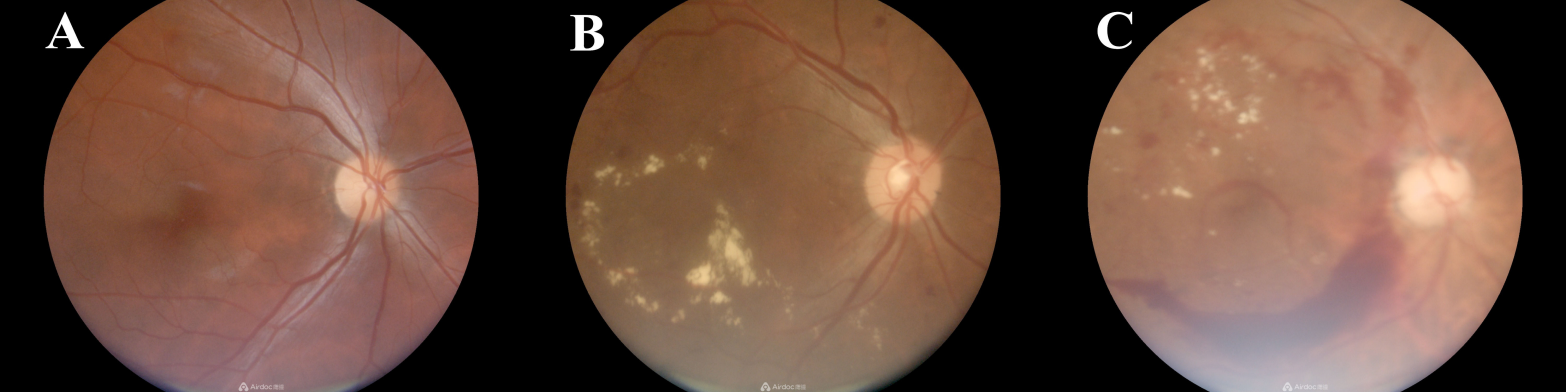
Photographs captured by non-mydriatic fundus camera of three types (**A**: normal control, **B**: non-proliferative DR, **C**: proliferative DR).

A total of 2327 T2DM patients were surveyed, comprising 1006 males and 1321 females. The median age was 69 years (interquartile range: 63–74 years), and the median duration of diabetes was 10.34 years (interquartile range: 4.69-14.03 years). 172 cases of DR were screened, yielding a prevalence rate of 7.39% (167 with moderate non-proliferative DR, 3 with severe non-proliferative DR, and 2 with proliferative DR). The photographs captured by non-mydriatic fundus camera of three types were shown in **Figure 1** (A: normal control, B: non-proliferative DR, C: proliferative DR). Specifically, there were 85 male and 87 female patients with DR. Among them, 148 were aged 60 and over, while 24 were under 60 years of age, with 1 individual younger than 30 years old. Notably, significant differences were observed between the T2DM group and the DR group in terms of diabetes duration, family history, history of hypoglycemia, glycated hemoglobin levels, sleep quality, MCI, hypertension, and eGDR distribution, as detailed in **Table 1**.

**Table 1:** Comparison of basic characteristics between two groups of T2DM patients.

Characteristic	Groups	Pure T2DM Group	DR Group	*χ*²	*P*
Age (years)		69 (63,74)	68 (63,73)	-1.026	0.305
Duration (years)		10.12 (4.36,13.08)	17.38 (10.40,27.91)	-10.627	<0.001
Gender (n,%)	Male	921 (91.55)	85 (8.45)	2.897	0.089
	Female	1234 (93.41)	87 (6.59)		
Education (n,%)	Elementary and below	732 (91.39)	69 (8.61)	2.768	0.251
	Junior/Senior high school	1272 (93.32)	91 (6.68)		
	College and above	151 (92.64)	12 (7.36)		
Marital Status (n,%)	Married	1836 (92.45)	150 (7.55)	0.079	0.779
	Unmarried	292 (92.99)	22 (7.01)		
Living alone (n,%)	Yes	211 (92.95)	16 (7.05)	0.043	0.835
	No	1944 (92.57)	156 (7.43)		
Residence (n,%)	Urban area	966 (93.42)	68 (6.58)	1.806	0.179
	Outside urban area	1189 (91.96)	104 (8.04)		
Smoking (n,%)	Yes	323 (91.76)	29 (8.24)	0.435	0.510
	No	1832 (92.76)	143 (7.24)		
Drinking (n,%)	Yes	217 (91.56)	20 (8.44)	0.423	0.516
	No	1938 (92.73)	152 (7.27)		
Family history (n,%)	Yes	931 (90.13)	102 (9.87)	16.727	<0.001
	No	1224 (94.59)	70 (5.41)		
Hypoglycemic history (n,%)	Yes	265 (88.63)	34 (11.37)	7.938	0.005
	No	1890 (93.20)	138 (6.80)		
Glycemic control (n,%)	Up to standard	1063 (95.00)	56 (5.00)	18.079	<0.001
	Not up to standard	1089 (90.37)	116 (9.63)		
HbA1c control (n,%)	Up to standard	1009 (97.30)	28 (2.70)	60.778	<0.001
	Not up to standard	1138 (88.77)	144 (11.23)		
Sleep disorder (n,%)	Yes	449 (90.16)	49 (9.84)	5.546	0.019
	No	1706 (93.28)	123 (6.72)		
Sedentary behaviour (n,%)	Yes	320 (92.75)	25 (7.25)	0.012	0.911
	No	1835 (92.58)	147 (7.42)		
Physical activity within target (n,%)	Yes	982 (92.64)	78 (7.36)	0.003	0.956
	No	1173 (92.58)	94 (7.42)		
Mild cognitive impairment (n,%)	Yes	759 (90.36)	81 (9.64)	9.734	0.002
	No	1396 (93.88)	91 (6.12)		
Hypertension (n,%)	Yes	1001 (91.33)	95 (8.67)	4.931	0.026
	No	1154 (93.74)	77 (6.26)		
Hyperlipidemia (n,%)	Yes	1767 (92.51)	143 (7.49)	0.142	0.707
	No	388 (93.05)	29 (6.95)		
eGDR (n,%)	Q1	686 (88.52)	89 (11.48)	32.623	<0.001
	Q2	723 (93.29)	52 (6.71)		
	Q3	746 (96.01)	31 (3.99)		

### 3.2 Association analysis between eGDR and DR

**Figure 2:**
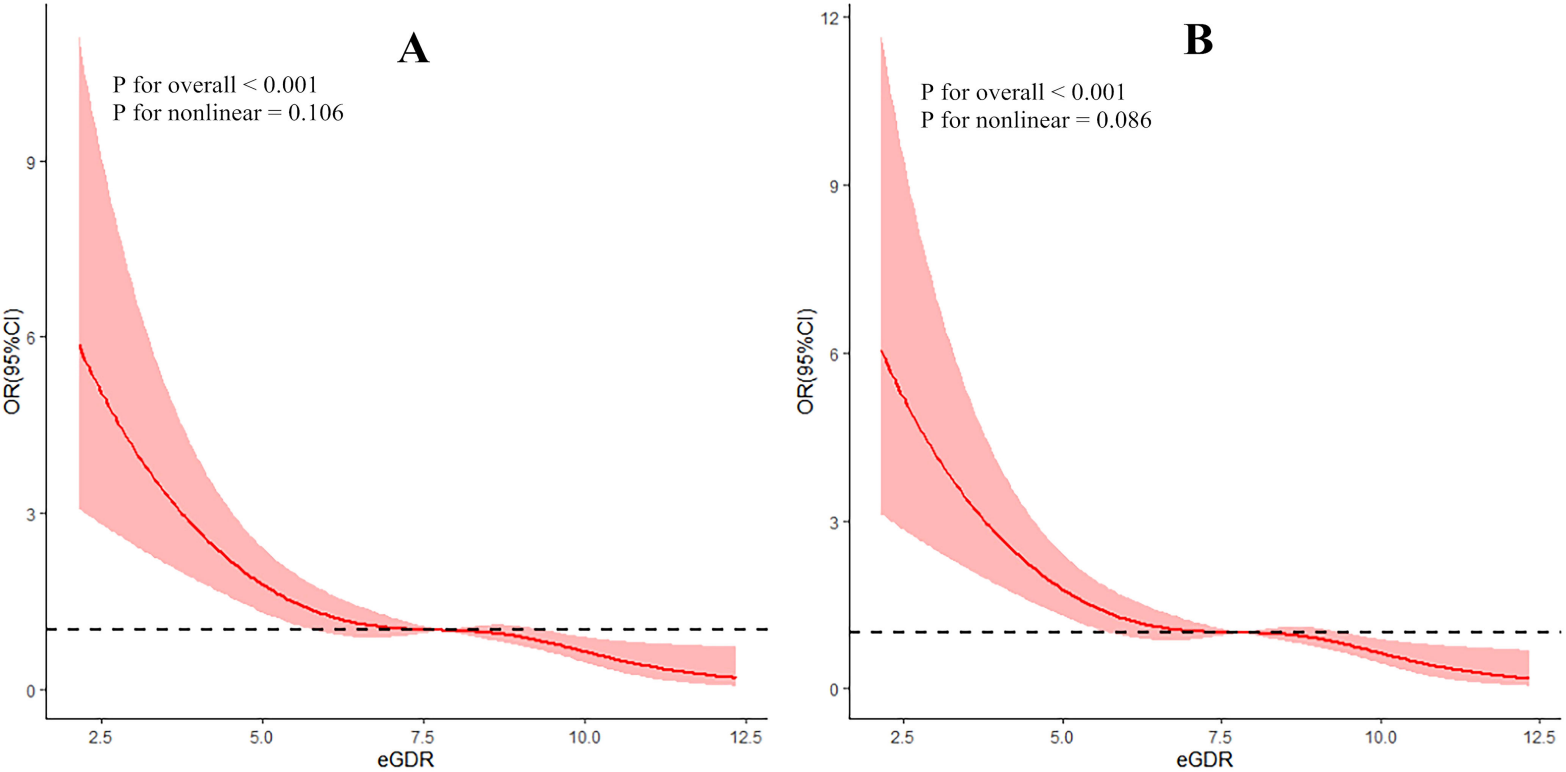
Dose-response relationship between eGDR and DR (**A**: without controlling covariates; **B**: with controlling covariates).

The RCS curve demonstrated a significant negative linear relationship between eGDR and DR (overall *P* < 0.05, non-linear *P* > 0.05), even after adjusting for covariates such as family history, history of hypoglycemia, sleep disorders, MCI, and disease duration, as illustrated in **Figure 2**.

Regression model 1 was constructed with T2DM with or without DR as the dependent variable and eGDR as the independent variable to analyze their association. The results showed that for each increase of one SD in eGDR, the risk of DR was reduced by 42.5%. Subsequently, variables found to be significant in univariate analysis (family history, history of hypoglycemia, sleep disorders, MCI, and disease duration) were included as covariates, leading to the construction of regression model 2. The findings revealed that for each increase of one SD in eGDR, the risk of DR was reduced by 40.4%. When eGDR was divided into tertiles, in Model 1, compared to the third tertile group, the risk of DR in the first tertile group was 3.122-fold higher, and in the second tertile group, it was 1.731-fold higher. After adjusting for covariates, the risk of DR in the first tertile group was 2.965 times that of the third tertile group, while in the second tertile group, it was 1.729 times that of the third tertile group (**Table 2**).

**Table 2:** Logistic regression results of the association between eGDR and DR.

eGDR	Cases	DR(n,%)	Model 1		Model 2	
			OR (95%*CI*)	*P*	OR (95%*CI*)	*P*
Continuous Variable						
For every one SD increase	2327	172 (7.39%)	0.575 (0.490,0.674)	<0.001	0.596 (0.504,0.704)	<0.001
Categorical variables						
Q1	775	89 (11.5%)	3.122 (2.048,4.760)	<0.001	2.965 (1.916,4.587)	<0.001
Q2	775	52 (6.7%)	1.731 (1.097,2.732)	0.018	1.729 (1.080,2.769)	0.023
Q3	777	31 (4.0%)	Ref.		Ref.	

### 3.3 Subgroup analysis

Subgroup analysis was conducted to assess the reliability of the regression association between eGDR and DR. Referring to previous studies, the subgroups included gender, age, smoking, drinking, and hyperlipidemia. The results in **Table 3** showed that the association between eGDR and DR was robust and reliable in these subgroups (*P* > 0.05 for all subgroups).

**Table 3:** Subgroup analysis of the association between eGDR and DR.

Subgroup	Groups	Cases	OR(95%*CI*)	*P*
Gender	Male	1006	0.587 (0.465,0.741)	0.375
	Female	1321	0.610 (0.481,0.773)	
Age	<60 years old	316	0.769 (0.513,1.153)	0.083
	≥ 60 years old	2011	0.564 (0.468,0.679)	
Smoking	Yes	352	0.549 (0.357,0.845)	0.957
	No	1975	0.603 (0.503,0.724)	
Drinking	Yes	237	0.527 (0.304,0.916)	0.616
	No	2090	0.599 (0.502,0.715)	
Hyperlipidemia	Yes	417	0.482 (0.309,0.749)	0.113
	No	1910	0.604 (0.503,0.725)	

### 3.4 Predictive effect of eGDR on DR

**Figure 3:**
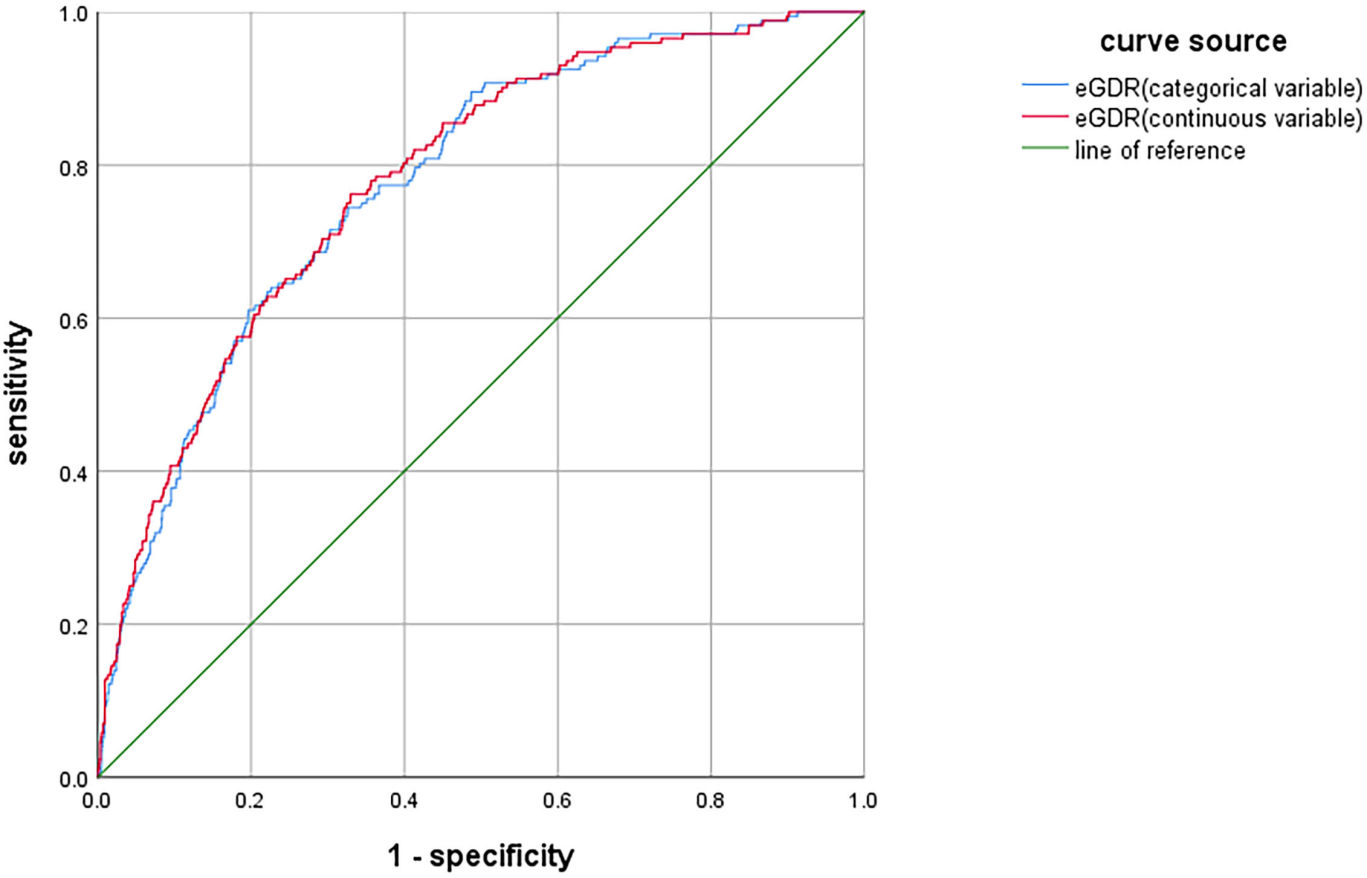
ROC curve analysis of eGDR for DR.

The ROC curve analysis was conducted to assess the diagnostic performance of eGDR in identifying DR. When eGDR was incorporated into the model as a continuous variable, the area under the curve (AUC) was 0.781 (95%*CI*: 0.747-0.815, *P* < 0.001). When eGDR was included as a categorical variable, the AUC was 0.777 (95%*CI*:0.743-0.811, *P* < 0.001), as illustrated in **Figure 3**. These results indicated that eGDR demonstrated substantial diagnostic accuracy for DR.

## 4 Discussion

Our study revealed a DR prevalence of 7.39% in Pudong New Area, and there was a significant negative linear relationship between eGDR and DR risk. Specifically, each standard deviation increase in eGDR was associated with a 40.4% reduction in DR risk.

With the increasing prevalence of T2DM, the disease burden of DR has escalated sharply by 25% over the past decade, leading to a continuous rise in visual impairment and associated care requirements (16). Research indicates that the accuracy of automated fundus examination for DR detection reaches 93%, with the performance of artificial intelligence systems being comparable to that of retinal experts in classifying DR (17, 18). In our study, the prevalence of DR was 7.39%, lower than that observed in northern cities in China, such as Harbin and Shijiazhuang (14, 15), potentially attributable to the ongoing integration of diabetes medical treatment and health management services in Shanghai (19), which has facilitated earlier detection and referral of DR patients. Although previous studies suggested that women exhibit a higher susceptibility to DR, and the association between eGDR and DR was more pronounced in this group (14, 20), no significant gender differences were identified in our investigation.

The screening of DR has been integrated into the community-based management of diabetic patients since 2018. Given the cognitive impairments associated with DR, along with the necessity for specialized fundus examination equipment and professional ophthalmologists to interpret the results, DR is frequently diagnosed at a moderate non-proliferative stage. Therefore, it is imperative to conduct a fundus examination immediately upon diagnosis of T2DM. Early intervention is particularly critical for patients with a family history of DR, a history of hypoglycemia, prolonged disease duration, poor glycemic control, hypertension, sleep disorders, and cognitive impairment to prevent vision loss.

IR represents a hallmark pathological characteristic in diabetic patients. The gold standard for quantifying IR is hyperinsulinemic-euglycemic clamp technique. However, this method is invasive and time-consuming, thereby limiting its applicability in clinical and epidemiological studies. Recently, eGDR, an index derived from waist circumference, glycated hemoglobin, and hypertension, has emerged as a reliable substitute indicator. Studies have demonstrated that eGDR exhibits comparable accuracy to the hyperinsulinemic-euglycemic clamping. Furthermore, some research suggests that eGDR may serve as a valuable tool for predicting and managing cardiovascular and cerebrovascular diseases (21). Our study identified a significant linear relationship between eGDR and DR. After adjusting for relevant confounders, for each increase of one SD in eGDR, the risk of DR is reduced by 40.4%. When eGDR was categorized into percentile groups, compared to the third percentile group, the risk of DR in the first percentile group was 2.965-fold higher, while the risk in the second percentile group was 1.729 times. These findings align with previous studies, which have indicated that obesity, poor glycemic control, and hypertension are risk factors for DR (22, 23). Subgroup analysis revealed that the association between eGDR and DR remained unaffected by factors such as age, gender, smoking, drinking, and hyperlipidemia, demonstrating high diagnostic efficacy for DR. Therefore, our study supports the use of eGDR as a target for prediction and intervention of DR. Recently, some studies have found that non-invasive and rapid examinations, such as skin autofluorescence, have certain predictive value for the early diagnosis of DR (24). It is expected that more convenient and reliable indicators will be discovered to evaluate the occurrence and progression of DR.

The limitations of this study are as follows: (1) This is a cross-sectional study, and the follow-up of patients without DR will be conducted to further validate these findings; (2) The utilization of an artificial intelligence system for screening may result in potential missed diagnoses, which could lead to an underestimation of the true prevalence of DR. (3) Self-reported questionnaires might introduce information bias due to recall inaccuracies. (4) The sampling survey methodology might introduce bias into the findings.

## 5 Conclusion

The prevalence of DR among T2DM in Pudong New Area was 7.39%, and there was a significant negative linear correlation between eGDR and the risk of DR. It is anticipated that eGDR could serve as a potential target for predicting and intervening in DR.

## Data Availability

The raw data supporting the conclusions of this article will be made available by the authors, without undue reservation.
